# A Deterministic Model to Quantify Risk and Guide Mitigation Strategies to Reduce Bluetongue Virus Transmission in California Dairy Cattle

**DOI:** 10.1371/journal.pone.0165806

**Published:** 2016-11-03

**Authors:** Christie Mayo, Courtney Shelley, N. James MacLachlan, Ian Gardner, David Hartley, Christopher Barker

**Affiliations:** 1 Department of Microbiology, Immunology, and Pathology, Colorado State University, Fort Collins, Colorado, United States of America; 2 Department of Pathology, Microbiology and Immunology, School of Veterinary Medicine, University of California Davis, Davis, California, United States of America; 3 Department of Health Management, Atlantic Veterinary College, University of Prince Edward Island, Prince Edward Island, Canada; 4 James M. Anderson Center for Health Systems Excellence, Cincinnati Children's Hospital Medical Center, Cincinnati, Ohio, United States of America; University of Texas Medical Branch at Galveston, UNITED STATES

## Abstract

The global distribution of bluetongue virus (BTV) has been changing recently, perhaps as a result of climate change. To evaluate the risk of BTV infection and transmission in a BTV-endemic region of California, sentinel dairy cows were evaluated for BTV infection, and populations of *Culicoides* vectors were collected at different sites using carbon dioxide. A deterministic model was developed to quantify risk and guide future mitigation strategies to reduce BTV infection in California dairy cattle. The greatest risk of BTV transmission was predicted within the warm Central Valley of California that contains the highest density of dairy cattle in the United States. Temperature and parameters associated with *Culicoides* vectors (transmission probabilities, carrying capacity, and survivorship) had the greatest effect on BTV’s basic reproduction number, *R*_*0*_. Based on these analyses, optimal control strategies for reducing BTV infection risk in dairy cattle will be highly reliant upon early efforts to reduce vector abundance during the months prior to peak transmission.

## Introduction

Bluetongue virus (BTV) is the cause of bluetongue (BT), an economically important, re-emerging arboviral disease of ruminants transmitted by various species of hematophagous *Culicoides* midges [1–3]. In North America, *Culicoides sonorensis (C*. *sonorensis)* is the predominant, if not exclusive, vector of BTV serotypes 10, 11, 13 and 17, which have long been endemic throughout extensive portions of the continent; BTV serotype 2 was first identified in Florida in 1982 and until recently was confined to the southeastern United States (US) [4–12]. Recent changes in the epidemiology of *Culicoides*-transmitted viruses, particularly the emergence of previously exotic viruses in Europe, have highlighted the dynamic nature of host-vector-pathogen interactions and implicated multiple environmental and anthropogenic factors as potential drivers of virus emergence and spread, including changes in climate, land use, trade and animal husbandry [12–15].

Until recently, the global distribution of BTV was relatively stable at temperate and tropical latitudes between approximately 40–50°N and 35–40°S [5]. However, the global distribution of BTV has recently and profoundly changed, with the invasion and spread of BTV throughout much of Europe, which previously had been free of the virus other than transient incursions into European countries bordering the Mediterranean Sea [16–18]. Recent experiences in Europe demonstrate the potentially devastating economic consequences of a BTV epidemic and the rapidly evolving epidemiology thereof—new species of insect vectors were involved in virus transmission, and in the case of BTV serotype 8 (BTV-8), the emergent virus was highly virulent to most species of domestic and non-African wild ungulates [19, 20]. Incursion of novel serotypes of BTV into historically endemic countries or regions including the southeastern US, Israel, Australia, Canada and California have also occurred recently [21–23], reflecting the diverse means, including wind-borne spread of vectors and livestock trade networks, that likely spread these viruses between regions [24, 25]. Climate change has been incriminated as a potential contributor to this recent expansion of the global distribution of BTV infection [26–29].

Since 2008, several mathematical models have been developed to better define the risk of BTV transmission and estimate the basic reproduction number (R_0_) within immunologically naïve livestock populations [30–34]. Much of the interest in modeling BTV transmission has arisen in response to the recent incursion of BTV-8 in Northern Europe; however, few of these models have based their predictions on contemporaneous data collected from field investigations in the study area of interest [30, 32, 35]. Furthermore, these models rarely incorporate realistic seasonal and spatial variability in vector-host ratios, or consider the discrepancy between trap catches when using traps baited with carbon dioxide versus light [10, 36]. With the exception of a recently modeled system in Alberta, Canada, where BTV infection does not occur currently, these models often assume that laboratory-derived parameters for vectors are solely applicable to modeled scenarios [13]. For example, many European models use laboratory-derived parameters for the North American vector, *C*. *sonorensis*, even though it is a species that naturally transmits strains and serotypes of BTV that are distinct from those in Europe [30, 32, 35]. Lastly, existing BTV transmission models have typically focused at spatial and temporal scales (1km–25km cells) that are not well suited to the study of macro -and micro-habitat factors associated with *Culicoides* activity, or to the individual farm scale at which control measures are usually applied.

Statistical models have been developed to identify seasonal environmental predictors of BTV infection within endemic regions [37–40]. However, mechanistic models that estimate the basic reproduction number (R_0_) have been used infrequently to quantify the risk of introduction of exotic BTV serotypes or virus strains [34]. BTV infection is endemic throughout much of California, distinctly seasonal, and BTV seroprevalence can range from 0–90% among adult dairy cattle [41]. Furthermore, the diversity of California’s landscape and dairy industry provide an excellent case study for the application of models to better understand environmental drivers of transmission dynamics of BTV infection among cattle [12, 42–44]. Recent epidemiological investigations in California have screened intensively-managed dairy cattle for BTV infections and concurrently estimated the abundance of *Culicoides* vectors in relation to their animal hosts by aspiration from sentinel animals [12, 41]. California is characterized by a Mediterranean climate with a diverse ecological landscape that is impacted by human activities, which include the intensive farming of crops and livestock. Cattle are held in high-density outdoor lots, and wastewater ponds, marshes, and irrigated fields associated with dairy cattle provide abundant larval habitats for *C*. *sonorensis* midges. Recent epidemiological field studies have provided detailed data for estimation of the host and vector state variables and transmission parameters utilized in mathematical models [10, 44]. Therefore, the goals of the present study were to 1) use mathematical modeling and field surveillance data to better characterize BTV transmission dynamics among intensively-managed dairy cattle in California, 2) define the geographic locations and seasonal windows at greatest risk for BTV transmission in California, and 3) identify key parameters that regulate transmission to inform control strategies for minimizing BTV infection risk in dairy cattle.

## Materials and Methods

### Data sources and parameter estimation

Data used for estimation of host, vector, and environmental states and parameters were obtained from both published literature as well as intensive surveillance studies that were conducted on four California dairy farms during 2009–2010, as previously described [12, 15, 41]. Briefly, *Culicoides* midges were captured weekly using CDC downdraft suction traps baited with CO2 (dry ice) [12, 32]. Serum and whole blood were collected from each cow and were analyzed for the presence of antibodies and viral RNA by BTV-specific competitive ELISA (cELISA; VMRD Inc., Pullman, WA) and quantitative RT-PCR (RT-qPCR) assays, respectively [45]. The work included animal research for field data and was approved by the University of California, Davis IACUC committee (approval number 16758).

Many of the vector parameters in the creation of our model were estimated directly from published studies of *C*. *sonorensis*, the primary vector of BTV within the western US (Table 1) [10, 11, 46–48]. Host and vector competence were assumed to be constant over time, and the model was evaluated for a typical herd of 1,000 dairy cows. Vector abundance and carrying capacity were defined by a regression model fitted to the seasonal pattern for relative abundance of *C*. *sonorensis* from field data from CO_2_-baited traps without UV light collected in 2009–2010 (S1 Fig) [12, 15]. Carrying capacity represents the maximum population size of a species that the environment can sustain given available resources [49]. This term cannot be measured directly, but trap counts provide an indication of the direction of population dynamics, with positive growth implying that carrying capacity is higher than abundance. Accordingly, the ratio of abundance to carrying capacity, k_V_, defines seasonal variation in birth and death rates, and for the purposes of the model, this ratio was defined for any particular date as the ratio of fitted abundance (N_V_) to the fitted abundance two weeks later (K_V_; Table 1).

**Table 1:** Parameter values for the model of bluetongue virus transmission.

Symbol	Description	Definition	References/Comments
b_H_	Birth rate of host	= d_H_	
b_V_	Birth rate of vector	= d_V_	
K_H_	Carrying capacity of host	1,000	Arbitrary
N_V_	Abundance of vector	= fitted trap counts	Fitted seasonal regression from field observations (S1 Fig)
K_V_	Carrying capacity of vector	= fitted trap counts (+ 14 days)	
1/d_V_	Life span of vector	7 days	[11]
1/d_H_	Life span of host	5 years	Typical lifespan of a California dairy cow
β_HV_	Adequate contact rate: host to vector	$=\frac{r_{HV}}{GP}$	
β_VH_	Adequate contact rate: vector to host	$=\frac{r_{VH}}{GP}$	[51]
r_HV_	Probability of successful BTV transmission from host to vector	0.02	[10, 52]
r_VH_	Probability of successful BTV transmission from vector to host	0.8	[53, 54]
1/ε_H_	Intrinsic incubation period of host	2 days	[11]
1/ε_V_	Extrinsic incubation period of vector as a function of temperature	= $\frac{1}{.0003T(T-10.4057)}$, T > 10.4100	[11]
1/γ_H_	Infectious period of host	60 days	[55]
GP	Gonotrophic period	$=\frac{2.7056}{.000171T(T-3.6966)(41.8699-T)},T>3.7$	[11]

Monthly and daily mean temperatures were obtained from PRISM Climate Group as 30-year climatic normals (1981–2010), gridded for California with a spatial resolution of 2.5 arcminutes (~ 4 km) [50]. Model results were visualized as a series of monthly maps and as daily time series for four representative locations chosen based on relevance for dairy farming and differences in seasonal temperature ranges: two cooler sites along the ocean (Eureka) and slightly inland (Petaluma), and two locations with hot summers in the northern Central Valley (Orland) and inland southern California (San Jacinto).

### Deterministic model

**Fig 1:**
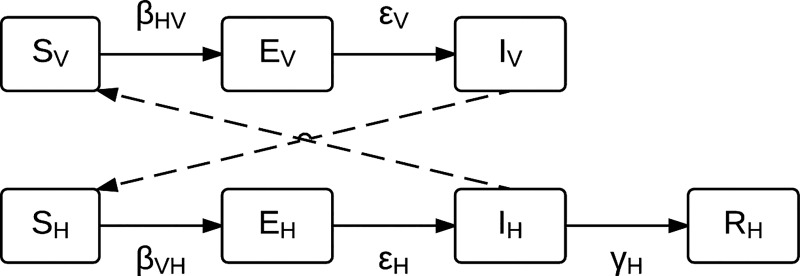
Flow diagram of the bluetongue virus (BTV) transmission model where host (cattle) compartments are denoted by the subscript H and vector (*Culicoides*) compartments are denoted by the subscript V. Each compartment is denoted by letter as susceptible (S), exposed (E), infected (I), or recovered (R). Refer to Table 1 for parameter definitions.

A compartmental, ordinary differential equation (ODE) model of BTV transmission was constructed. The model is based on a single host (cattle) and a single vector (*C*. *sonorensis*), which is the predominant vector of BTV on California dairy farms, and is depicted schematically in Fig 1. For the purpose of this model, only the adult vector population was modeled, and the vector population was able to transmit BTV to hosts but not through vertical transmission to their offspring, consistent with recent findings [56]. Once infectious, *Culicoides* vectors remained infectious for the remainder of their lifespan. Infection was assumed not to affect *Culicoides* behavior or longevity. Cattle hosts (denoted by the subscript H) became infected when fed upon by infectious *Culicoides* vectors (denoted by the subscript V) and then recovered, developing lifelong immunity to re-infection. Transmission of a single BTV serotype was considered within the context of this model. Populations of cattle and *Culicoides* midges contained susceptible (S_i_), incubating (infected but not yet infectious, E_i_), and infectious (I_i_) individual animals. Infected cattle recovered from infection with immunity (R_H_), and adult *Culicoides* were assumed to emerge uninfected. The system of ODEs is given below:

For dairy cattle hosts (see Table 1 for a description of notation),

$$\begin{array}{l} \frac{{dS}_{H}}{dt}=b_{H}N_{H}-\beta_{VH}S_{H}\frac{I_{V}}{N_{V}}\\ \frac{{dE}_{H}}{dt}=\beta_{VH}S_{H}\frac{I_{V}}{N_{V}}-\varepsilon_{H}E_{H}-d_{H}E_{H}\frac{N_{H}}{K_{H}}\\ \frac{{dI}_{H}}{dt}=\varepsilon_{H}E_{H}-\gamma_{H}I_{H}-d_{H}I_{H}\frac{N_{H}}{K_{H}}\\ \frac{{dR}_{H}}{dt}=\gamma_{H}I_{H}-d_{H}R_{H}\frac{N_{H}}{K_{H}}\\ \frac{{dN}_{H}}{dt}=N_{H}\left(b_{H}-d_{H}\frac{N_{H}}{K_{H}}\right) \end{array}$$

For adult *Culicoides* vectors,

$$\begin{array}{l} \frac{{dS}_{V}}{dt}=\frac{b_{V}K_{V}}{N_{V}}N_{V}-\beta_{HV}S_{V}\frac{I_{H}}{N_{H}}-d_{V}S_{V}\frac{N_{V}}{K_{V}}\\ \frac{{dE}_{V}}{dt}=\beta_{HV}S_{V}\frac{I_{H}}{N_{H}}-\varepsilon_{V}E_{V}-d_{V}E_{V}\frac{N_{V}}{K_{V}}\\ \frac{{dI}_{V}}{dt}=\varepsilon_{V}E_{V}-d_{V}I_{V}\frac{N_{V}}{K_{V}}\\ \frac{{dN}_{V}}{dt}=\frac{b_{V}K_{V}}{N_{V}}N_{V}-d_{V}N_{V}\frac{N_{V}}{K_{V}} \end{array}$$

In order to calculate the basic reproduction number (R_0_) for horizontal (vector-borne) transmission, the exposed and infectious compartments of cattle and *Culicoides* vectors were evaluated. The analytical expression for R_0_ was computed by applying the method previously described [57]. To simplify the computation of R_0_, system equations were normalized to consider the percent of the population made up by each compartment:

$$\frac{d}{dt}\left[\begin{array}{c} E_{H}\\ I_{H}\\ E_{V}\\ I_{V} \end{array}\right]=F-V=\left[\begin{array}{c} \beta_{VH}S_{H}I_{V}\\ 0\\ \beta_{HV}S_{V}I_{H}\\ 0 \end{array}\right]-\left[\begin{array}{c} \varepsilon_{H}E_{H}+d_{H}k_{H}E_{H}\\ -\varepsilon_{H}E_{H}+\gamma_{H}I_{H}+d_{H}k_{H}I_{H}\\ \varepsilon_{V}E_{V}+d_{V}k_{V}E_{V}\\ -\varepsilon_{V}E_{V}+d_{V}k_{V}I_{V} \end{array}\right]$$

where $k_{H}=\frac{N_{H}}{K_{H}},k_{V}=\frac{N_{V}}{K_{V}}$.

The next generation matrix, FV-1 is then calculated, with

$$F=\left[\frac{{\partial F}_{i}}{{\partial x}_{j}}(x_{0})\right]\;and\;V=\left[\frac{{\partial V}_{i}}{{\partial x}_{j}}\;(x_{0})\right],\;1\leq i,j\leq m,$$

where x0 = disease-free equilibrium (DFE).

$$\begin{array}{l} F=\left[\frac{{\partial F}_{i}}{{\partial x}_{j}}(x_{0})\right]=\left[\begin{array}{cccc} 0 & 0 & 0 & \beta_{VH}\\ 0 & 0 & 0 & 0\\ 0 & \beta_{HV} & 0 & 0\\ 0 & 0 & 0 & 0 \end{array}\right]\\ V=\left[\frac{{\partial V}_{i}}{{\partial x}_{j}}(x_{0})\right]=\left[\begin{array}{cccc} \varepsilon_{H}+d_{H}k_{H} & 0 & 0 & 0\\ -\varepsilon_{H} & \gamma_{H}+d_{H}k_{H} & 0 & 0\\ 0 & 0 & \varepsilon_{V}+d_{V}k_{V} & 0\\ 0 & 0 & -\varepsilon_{V} & d_{V}k_{V} \end{array}\right] \end{array}$$

R_0_ is defined as the spectral radius of the next generation matrix, FV^-1^

$$R_{0}=\rho ({FV}^{-1})=\sqrt{\left(\frac{\varepsilon_{V}}{\varepsilon_{V}+d_{V}k_{V}})(\frac{\varepsilon_{H}}{\varepsilon_{H}+d_{H}k_{H}})(\frac{\beta_{VH}}{d_{2}k_{V}})(\frac{\beta_{HV}}{\gamma_{H}+d_{H}k_{H}}\right)}$$

The first term in the R_0_ equation corresponds to the vector-borne transmission; R_0_ is comprised of two parts corresponding to *Culicoides*-cattle interactions. The term $\left(\frac{\varepsilon_{V}}{\varepsilon_{V}+d_{V}k_{V}}\right)$ represents the probability of an adult *Culicoides* midge surviving the extrinsic incubation period to the point where they can become infectious. Similarly, the term $\left(\frac{\varepsilon_{H}}{\varepsilon_{H}+d_{H}k_{H}}\right)$ is the probability that cattle survive to the point where they are infectious. The term $\left(\frac{\beta_{VH}}{d_{H}k_{V}}\right)$ represents the mean number of bites *Culicoides* make throughout the course of their lifetime, and the term $\left(\frac{\beta_{HV}}{\gamma_{H}+d_{H}k_{H}}\right)$ represents the mean number of times cattle are bitten by *Culicoides* midges during the time these vectors are infectious.

### Sensitivity analysis

Latin hypercube sampling was used to test the sensitivity of the model to each input parameter, as used in previous studies [30, 32]. For each parameter, a uniform distribution was assigned (see Table 2). The system was solved numerically using a large set (n = 200) of sampled model parameters, and the following parameters were evaluated for their influence on R_0_: k_V_, r_HV_, r_VH_, GP, N_H_, K_H_, d_H_, γ_H_, ε_H_, d_V_, and EIP. Partial rank correlation coefficients were used to assess the strength and significance of associations between parameters and R_0_ values.

**Table 2:** Ranges of parameters and partial rank correlation coefficients (PRCC) and 95% confidence intervals in relation to R0.

Symbol	Range	PRCC	95% CI
k_V_	[0, 10]	-0.7385	[-0.7799, -0.6996]
r_HV_	[0.01, 0.1]	0.3769	[0.3146, 0.4245]
r_VH_	[0.1, 1]	0.3355	[0.2842, 0.4040]
GP	[0, 50]	-0.7555	[-0.7801, -0.7235]
N_H_	[100, 10 000]	-0.0488	[-0.1101, 0.0066]
K_H_	[100, 10 000]	0.0339	[-0.0253, 0.1045]
d_H_	[0.0001, 0.001]	-0.0002	[-0.0532, 0.0675]
γ_H_	[0.005, 0.05]	-0.3467	[-0.3983, -0.2912]
ε_H_	[0, 1]	-0.0004	[-0.0544, 0.0646]
d_V_	[0.01, 1.0]	-0.7102	[-0.7491, -0.6702]
EIP	[1, 50]	-0.4306	[-0.4789, -0.3624]

## Results

### Geographic limits

**Fig 2:**
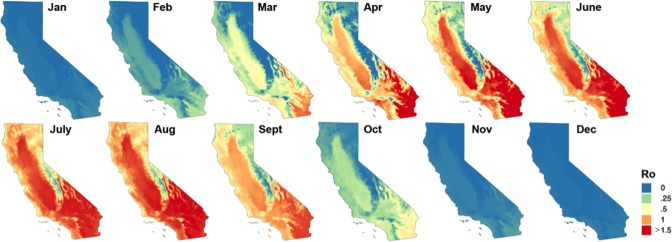
Monthly predicted spatial distribution of bluetongue virus (BTV) among dairy cattle in California. The map shows the calculated reproductive ratios, R0, projected based on 30-year mean temperatures, 1981–2010.

The highest R_0_ values (1.70 to 2.30) were in inland areas of southern California and the Central Valley (Fig 2), which have intensively managed dairies and hotter summers and cooler winters than coastal regions of California. Remaining portions of the state had a heterogeneous distribution of R_0_ values ranging from 0.03 to 1.72.

**Fig 3:**
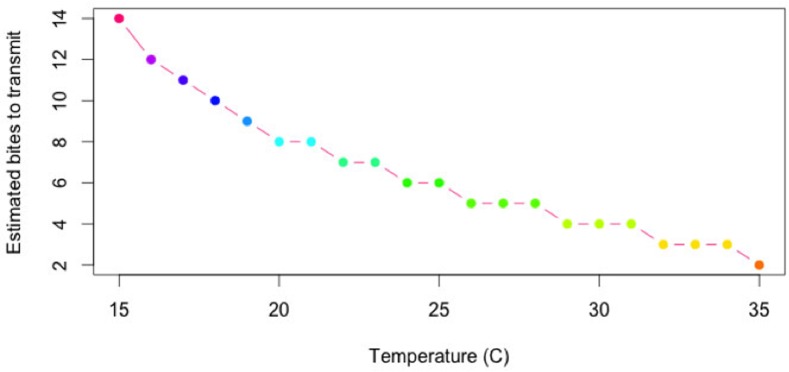
Estimated bites required for transmission of bluetongue virus (BTV) by *C*. *sonorensis* at varying temperatures. These estimates are derived from laboratory data used to estimate extrinsic incubation period and gonotrophic period as a function of temperature.

A second metric for transmission efficiency, the number of vector bites required for BTV transmission following vector infection, was derived from temperature-dependent estimates of the extrinsic incubation period (EIP) and gonotrophic period (GP) in *Culicoides* vectors. The estimated number of bites from infection to transmission was equal to the EIP divided by GP (Fig 3).

### Seasonality

**Fig 4:**
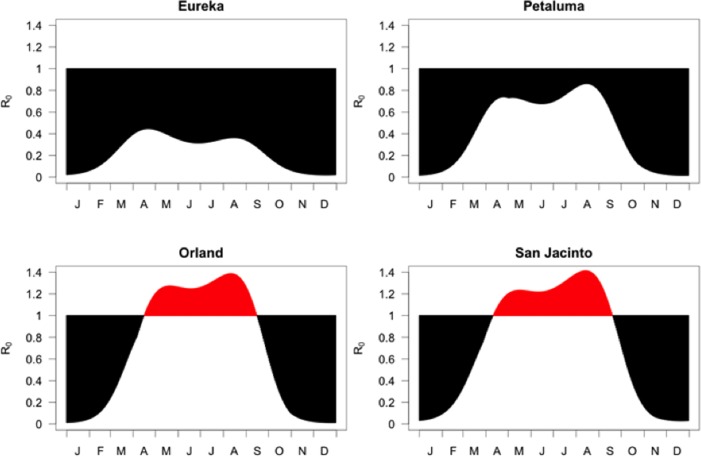
Seasonal patterns in basic reproduction ratios, R_0_, four representative locations in California based on 30-year mean temperatures. Locations include two cooler sites along the ocean (Eureka) and slightly inland (Petaluma), as well as representative locations with hot summers in the northern Central Valley (Orland) and inland southern California (San Jacinto).

Seasonal patterns for R_0_ were evaluated for representative locations in California (Fig 4). In all areas, R_0_ exhibited two peaks, with the first associated with early population growth of female *C*. *sonorensis*, and the second associated with high temperatures and peak *C*. *sonorensis* abundance in mid-late summer. The predicted period of sustained BTV transmission (R_0_ > 1) began in April and ended in September in both the Sacramento Valley (Orland) and southern California (San Jacinto). In cooler maritime areas of northern California, R_0_ values remained well below 1 throughout the year. R_0_ values were universally low throughout all regions of California from Nov through Feb.

### Sensitivity analysis

Sensitivity analysis revealed that R_0_ was negatively correlated with the ratio of total midge population to vector carrying capacity (k_V_, defined as N_V_/K_V_) and vector death rate (d_V_), such that an increase in either of these values corresponds with a decrease in R_0_. R_0_ was positively correlated with vector and host competence, r_HV_ and r_VH_, and biting rates (1/GP) (Table 2). R_0_ was negatively correlated with extrinsic incubation periods (EIP) in the vector. R_0_ values were negatively correlated with faster host recovery from infection (shorter infectious periods), but were not sensitive to variations in parameter values pertaining to life histories of cattle, including mortality, cattle numbers, or carrying capacities.

## Discussion

There is compelling evidence that the global distribution of BTV in different ecosystems is rapidly and dramatically changing so that the virus is spreading beyond its traditional boundaries in North America, Europe, and elsewhere, likely in part as a consequence of climate change [26, 58]. Mathematical modeling approaches have been utilized during the recent European epidemic to provide predictive estimates of risk, but these models have been based largely on historical and exogenous data that may not reflect the current situation or the particular ecosystem [59]. The model presented in this paper also has some of these limitations but data collected from intensive surveillance of California dairy cattle during 2009–2010 inform and validate a relevant model for predicting risk among dairy cattle in an endemic region.

This study focused on intensively raised dairy cattle as the host species because they comprise the largest population of livestock within the state (~2 million)[60], and the density of other ruminant species in the immediate vicinity of dairy farms is typically low. Cattle are highly competent hosts for BTV and are frequently bitten by *C*. *sonorensis*, and our results indicated that R_0_ was insensitive to the number of hosts over the broad range of herd sizes we considered (100 to 10,000 cattle). This also suggests that additional hosts adjacent to the herd would be unlikely to alter our estimates of transmission if their role in BTV transmission is similar to that of cattle. However, further study is warranted to understand the spatial dynamics of BTV transmission within and among herds and whether hosts in neighboring areas could contribute to BTV transmission. If additional hosts are present, whether they would dilute or potentiate transmission to cattle would depend on their relative competence and the degree to which they are fed upon by *C*. *sonorensis*.

Seroprevalence for BTV has been shown to vary widely among dairy cattle herds in California and elsewhere, with estimated values from 0 to 90%[12, 39, 43, 44]. Our R_0_ estimates of 1.7–2.3 in the most intensively managed dairy regions of California suggest that seroprevalence values in adult cattle would be expected to lie around the middle of the observed range, although precise expectations are complicated by the seasonality of BTV. Our highest R_0_ estimate would not be expected to result in a seroprevalence as high as 90%, especially given the limited seasonal window for transmission. This difference is attributed in part to our use of long-term temperature averages and fitted *C*. *sonorensis* abundance patterns, which smooth the variation in these quantities. Within an individual season or on an individual farm, it is possible that R_0_ values could be higher, either due to more favorable short-term conditions for transmission than we captured with long-term averages, stochasticity that is not represented by our deterministic model, or in some areas, unrecognized BTV-competent wild ruminants that could serve as a source for transmission to cattle [61, 62].

This model assesses the risk of BTV infection among a population utilizing a quantitative framework by calculating the basic reproduction number (R_0_) derived from vector, host, and virus parameters [30]. A defining feature of this model, in comparison with other vector-borne disease models, is the incorporation of temperature dependence and the use of laboratory and field observations of direct relevance to the cattle-*C*. *sonorensis* system to define parameters for biting rate, extrinsic incubation period (EIP), and vector dynamics. In particular, carrying capacity has been used historically in ecological modeling but has been largely ignored in vector-borne disease modeling systems [63–65]. Based on the strong correlation of this parameter with R_0_, carrying capacity and the ecological drivers of this equation may be important variables to consider in future vector-borne disease modeling scenarios.

Higher rates of transmission between vectors and hosts were associated with higher R_0_ values. Transmission rates were the product of temperature-dependent biting rates and vector and host competence. For BTV, vector competence is typically low as compared to other vector-borne diseases, but this is offset by the very large number of *Culicoides* per host. Parameters with a broad range of variability, such as vector carrying capacity, mortality, and biting rates would be expected to be stronger drivers of changes in R_0_. Our definition of 60 days for the infectious period of cattle factors the extended RNAemia that occurs in BTV-infected cattle and, therefore, should be regarded as a maximal estimate with respect to this parameter,[55, 66]. An earlier modeling study used a value of 20.6 days [30]. Our analyses show, as expected, that R_0_ is sensitive to this choice and shorter duration of estimated infectious viremia would reduce R_0_.

Both temperature and vector habitat availability are important drivers of seasonal and geographic variation in BTV risk, respectively. Temperature broadly influences transmission through several parameters in the model and plays an important role in driving the onset of a seasonal BTV transmission cycle (transmission rates and EIP), whereas land use and vegetation most likely contribute to the establishment of an ecosystem conducive for a thriving vector population (carrying capacity).

Utilizing parameters obtained from the literature and our sentinel surveillance program, this model pointed to the Central Valley and southeastern deserts of California as the areas at greatest risk of BTV transmission. This finding is important because of the large numbers of cattle combined with high temperatures and other agricultural practices (i.e. irrigation) that are conducive to seasonal (May-November) *Culicoides* activity in these areas. Additionally, north and central eastern portions of the Central Valley of California contain large tracts of federal land grazed by livestock; therefore, it is possible contact between deer and livestock could explain the greater risk of BTV infection that occurs in this region. It is suspected that there is a decreased risk of BTV infection within the southeastern, northwestern, and northeastern portions of California due to extreme temperatures that occur in those areas, both hot and cold depending on region. Seasonally in 2010, the model demonstrated that the initiation of predicted BTV infection prevalence of dairy cattle was closely associated with the initial collections of female *C*. *sonorensis* midges. The predictions of the model most likely reflect the sensitivity of the R_0_ calculation to temperature; therefore, these R_0_ values should be considered as relative indicators of risk rather than absolute thresholds. However, the time lag from initiation of predicted BTV transmission in dairy cattle (April) to peak values (August) indicates that preventive control measures for minimizing the seasonal amplification of BTV may be most effective if they are initiated up to 3.5 months prior to the peak of infection in dairy cattle. In combination with the sensitivity analysis, the spatial and seasonal results indicate that simple and cost-effective strategies to reduce vector abundance (i.e. disrupting larval habitat) might be the most efficacious mitigation strategy to decrease BTV transmission among dairy cattle within California.

Before recommending these strategies for control or prevention, it is important to acknowledge three important limitations in this model. First, the assumption of a single cattle population does not address the risk of disease spread due to animal movement. Second, vector (*Culicoides*) dispersal is not accounted for in the model and it has been demonstrated that these insects can fly up to 6 km in 30 hours against prevailing winds [24, 67]. Third, overwintering mechanisms are not addressed in the model, leaving little understanding about interannual maintenance of the virus in the host or the vector population [68, 69]. In addition, the model is highly reliant on sentinel dairy cattle parameters obtained in California so future studies should include other ruminant species. Other models that have been developed in Europe elaborate on various potential (and biologically uncertain) latent stages in the animal hosts of BTV, and such models might thus provide more accurate prediction of epidemics [32]. However, these models rely heavily on speculation and the data available from the BTV-8 outbreaks in Europe during 2006 and 2007 that are not relevant to the mechanism of overwintering of BTV in California [56, 70]. Clearly, appropriate consideration of relevant parameter values is necessary to apply those values to models developed for accurately predicting transmission dynamics within the US and elsewhere.

The model presented in this paper is a simplified representation of a complex biological system that cannot be completely reproduced or predicted. Thus, as with all models, much of the value is within the process of building and interpreting it. Ultimately, the purpose of most modeling is to generate information that can guide effective policy or mitigation strategies for control and prevention of disease [32, 71]. Appropriate mitigation strategies in endemic regions are likely different from those previously described for epidemics of BTV infection in immunologically naïve livestock populations, such those that occurred recently in Europe [32, 71, 72]. At least four strategies could be utilized to reduce BTV infection on intensive dairy farms: 1) application of insecticide to reduce populations of adult *C*. *sonorensis* midges; 2) reduced usage of lagoon wastewater ponds at each farm to limit habitat for *C*. *sonorensis* larvae; 3) vaccination of cattle to prevent BT and/or BTV infection; and 4) culling of BTV-infected cattle and/or restriction of movement of potentially virus-infected cattle [66, 73–76]. Therefore, while it remains difficult to recommend a single control measure for such a complicated transmission cycle, models based on relevant and comprehensive data provide a better understanding of risk for BTV infection transmission so that the most appropriate control strategy can be implemented [77].

## Supporting Information

S1 Fig Fitted seasonal pattern for relative abundance of *Culicoides sonorensis* per CO_2_-baited trap-night. (TIF)
