# Supplementary figures and images for: A Deterministic Model to Quantify Risk and Guide Mitigation Strategies to Reduce Bluetongue Virus Transmission in California Dairy Cattle
Source: PLoS One. 2016 Nov 3;11(11):e0165806. doi: 10.1371/journal.pone.0165806 (PMC5094782; doi:10.1371/journal.pone.0165806)

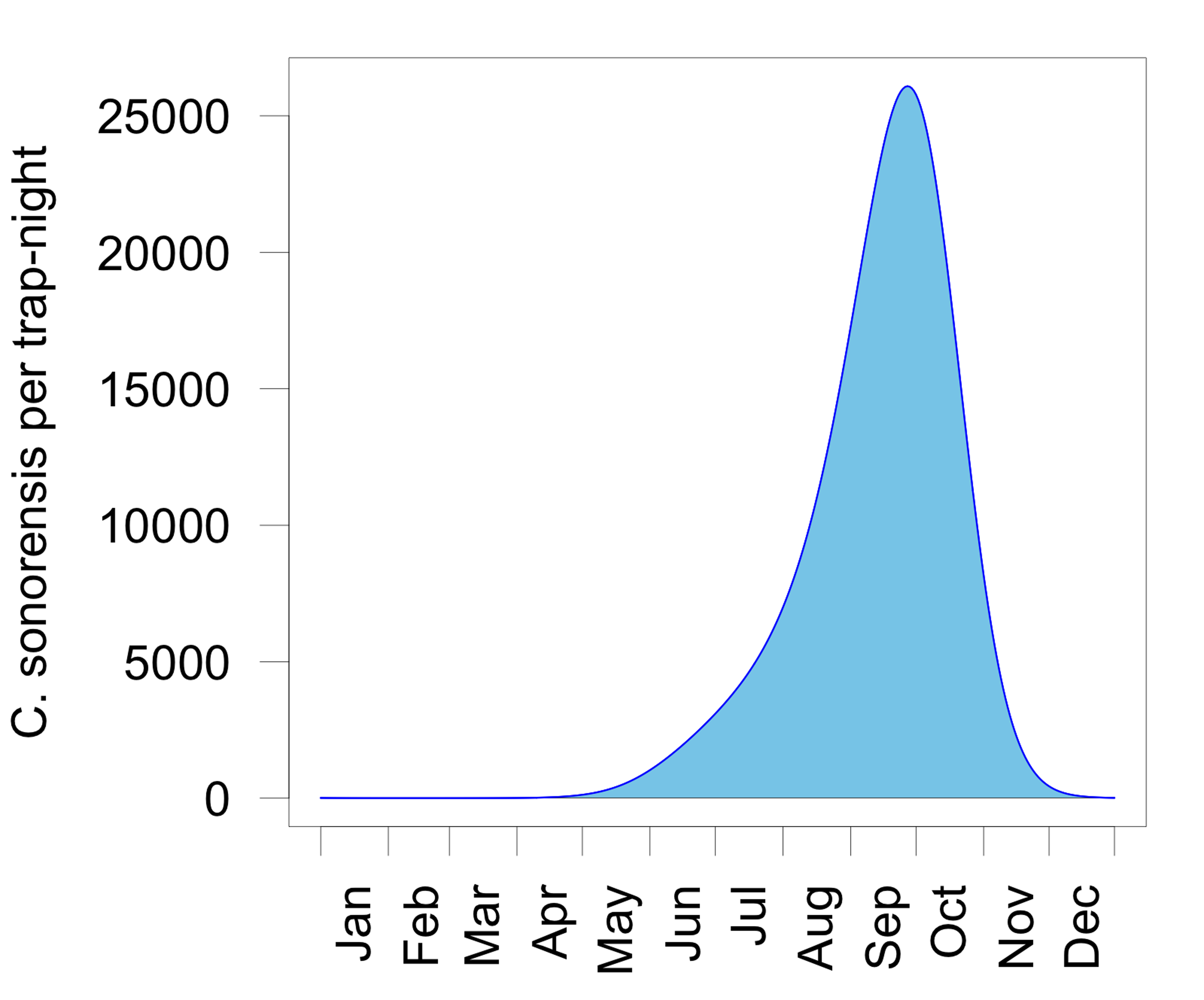

Supplement: S1 Fig — (TIF) [file pone.0165806.s001.tif]
